# Risk factors of serofast state in patients undergoing syphilis: a meta-analysis of 17 cohort studies

**DOI:** 10.3389/fimmu.2025.1689904

**Published:** 2025-12-05

**Authors:** Xin Zeng, Yuan Ouyang, Haoyun Wang, Linna Liu, Jiaxin Chen, Chengbin Zhu, Hong Huang, Jin Lin, Yanan Niu, Lan Liao, Na Yang, Chunlan Xiao, Weidong Gong, Peng Liu

**Affiliations:** 1 Institute of Pathogenic Biology, Basic Medical School, Hengyang Medical School, University of South China, Hengyang, China; 2 Affiliated Hengyang Hospital of Hunan Normal University &Hengyang Central Hospital, Hengyang, Hunan, China

**Keywords:** syphilis, treponema pallidum, serofast state, serological diagnosis, sexually transmitted disease

## Abstract

**Objective:**

The serofast state in syphilis refers to a persistent serological status where patients maintain stable specific antibody titers despite receiving standardized anti-syphilitic therapy, whose underlying mechanisms remain incompletely elucidated. This study aims to systematically identify risk factors associated with the occurrence of syphilitic serofast state through comprehensive clinical data analysis.

**Method:**

We performed a systematic literature search in PubMed and Embase databases for studies published up to February 20, 2025. A random-effects model was employed for meta-analysis, with effect estimates expressed as relative risk (RR) presented with 95% confidence intervals (CIs). Methodological evaluations including sensitivity analyses and publication bias assessment were conducted to assess result robustness.

**Results:**

A total of 17 cohort studies involving 4,662 eligible participants were included in this meta-analysis. The pooled results demonstrated significant associations between serofast state and primary syphilis (RR = 0.65; 95% CI: 0.44-0.96), latent syphilis (RR = 2.14; 95% CI: 1.47-3.11), female gender (RR = 1.19; 95% CI: 1.01-1.41), HIV coinfection (RR = 1.40; 95% CI: 1.09-1.79), and lower rapid plasma reagin (RPR) titers (≤1:32) (RR = 1.47; 95% CI: 1.02-2.13). No statistically significant associations were observed for secondary syphilis (RR = 0.81; 95% CI: 0.59-1.12), age >40 years (RR = 1.41; 95% CI: 0.80-2.49), or benzathine penicillin treatment (RR = 0.99; 95% CI: 0.86-1.13). These findings were validated through leave-one-out sensitivity analysis.

**Conclusion:**

Female gender, HIV coinfection, primary syphilis, latent syphilis, and low rapid plasma reagin (RPR) titers (≤1:32) emerged as significant risk factors for serofast state development, requiring particular attention during therapeutic management to optimize syphilis treatment outcomes.

## Introduction

Syphilis is a sexually and vertically transmitted bacterial infection caused by the spirochete *Treponema pallidum (*1–3), which invades multiple systemic organs, leading to clinical manifestations such as neurosyphilis, ocular syphilis, and cardiovascular syphilis, severely endangering human health (4–6). The incidence of syphilis is increasing and remains a global public health issue with the latest estimates from the World Health Organization that approximately 17.7 million adults aged 15 to 49 were infected with syphilis globally in 2012 and an estimated 6.3 million new cases occurred in 2016 (7–10).

Penicillin is the primary drug for the treatment of syphilis (11, 12). Most patients experience significant improvement in clinical symptoms and serological indicators after standard treatment (13, 14). However, not all patients achieve serological reversal following recommended therapy. In a small number of cases, after adhering to anti-syphilis treatment, the non-treponemal antibody titers decrease to a certain level, cease to decline further, and remains within a specific titer range for a long time. This phenomenon is known as “syphilis serofast” (15–19). This clinical phenomenon has sparked considerable debate: whether the persistence of serological positive reactions indicates the presence of an active infectious focus within the body, or only represents an immunological memory response post-treatment. It is noteworthy that serofast state is significantly correlated with the progression of neurosyphilis (20). Currently, there is no unified understanding globally regarding the epidemiological characteristics, pathogenesis, and clinical management strategies for syphilis serofast, making it an important issue that urgently needs to be addressed in the field of syphilis treatment.

Currently, there are two commonly used definitions of serofast state based on the decline in non-treponemal antibody titers: a decline of no more than twofold or no more than fourfold. After considering factors such as the need to balance sensitivity and specificity, ensuring reliable classification of serofast state while maintaining clinical applicability, as well as the consistency of results across studies and the clinical relevance of antibody titer thresholds, we selected the fourfold decline (equivalent to a twofold dilution) as the standard for defining serofast state. Specifically, we define serofast state as a less than fourfold (twofold dilution) reduction in non-treponemal antibody titer between 6- and 12-months post-treatment, or as the persistence of a low titer beyond this period.

Syphilis serofast poses a significant challenge in clinical diagnosis and treatment, exerting a dual threat to both the physical and mental health of patients. This clinical conundrum not only increases the diagnostic and therapeutic difficulties for healthcare providers but also imposes substantial psychological stress on patients. This study systematically analyses the factors influencing the serofast state, aiming to elucidate the characteristic patterns of serological responses. The findings are expected to provide a theoretical foundation for establishing early warning mechanisms and optimizing treatment protocols, and enhance clinicians’ understanding of the serofast phenomenon, thereby improving patient prognosis and alleviating their psychological distress.

## Methods

### Standard protocol approvals and registrations

This study was conducted and reported in accordance with the Cochrane Handbook and the PRISMA (Preferred Reporting Items for Systematic Reviews and Meta-Analyses) 2020 guidelines (21). The review was registered in PROSPERO with the registration unique identifying number (UIN) of CRD420250650375.

### Search strategy

Without restrictions on language or publication year, two independent authors searched PubMed, Embase from their inceptions to February 20,2025 for articles about the factors associated the syphilis serofast state. We combined free words related to syphilis and serodiagnosis, used Medical Subject Headings (MESH) to search PubMed, and used Embase topic entries (Entree) to search Embase. The search terms used syphilis or “*T. pallidum*” or “Syphilis Serodiagnosis” or “Drug Therapy” or “Treatment Outcome”. In addition, to find potentially relevant articles, the studies cited in relevant reviews and systematic reviews were manually searched. When the queues described in the articles were the same, we kept only the most recent articles or those with the largest sample size. The search strategy used for the databases is presented in **Supplementary Table S1**.

### Selection criteria

The study was performed in accordance with the PECOS guidelines (22). Two reviewers independently assessed the full texts of the studies to determine their eligibility according to the selection criteria, and prospective or retrospective cohort studies involving the factors associated the syphilis serofast state were included.

Only studies that meet the following criteria will be included: (1) Participants: Patients underwent syphilis; (2) Exposure: Risk factors associated with the incidence of serofast state, such as sex, age, HIV-positive, Benzathine penicillin use or syphilis stage; (3) Comparator: comparison group with lower exposure or no exposure to a modifiable risk factor; (4) Outcome: risk of serofast state, presented as the odds ratio (OR) with the corresponding 95% CI; (5) Study design: prospective or retrospective cohort study.

The exclusion criteria include the lack of available full-text articles, reviews, letters, comments, or conference abstracts. We also excluded studies that did not include syphilis or did not report sufficient data to investigate serum fixation factors.

### Data extraction

The following basic characteristics were retrieved from each article: first author, year of publication, type of study, place of origin of patients, sample size, observation period, average age, syphilis stage, the definition, and risks of serofast state (**Table 1**). The two evaluators independently extracted data from the selected literature, recorded the relevant data in the designed data extraction form, evaluated the qualifications of these articles again, and Any disagreement was resolved through discussion with a senior author.

**Table 1:** Characteristics of studies included in meta-analysis.

Trail	First author	Year	Study design	Region	Observation period	Sample size	Average age (years)	Male%	Primary syphilis
1	Cai, S. N (20).	2017	Retrospective	China	2008-2016	402	33	35.32%	NR
2	Leeyaphan, C (27).	2019	Retrospective	Thailand	2011-2015	179	31.9	97.20%	6
3	Liu, J (28).	2022	Prospective	China	2015-2018	445	33.22	35.28%	5
4	Liu, Y (29).	2020	Retrospective	China	2009-2016	114	36	39.50%	10
5	Marchese, V (30).	2022	Retrospective	Italy	2013-2021	382	38	77.00%	0
6	Pastuszczak, M (31).	2019	Prospective	Poland	2016-2018	80	32.65	93.75%	0
7	Pastuszczak, M (32).	2019	Prospective	Poland	2015-2017	48	32.79	93.75%	0
8	Pastuszczak, M (33).	2018	Retrospective	Poland	2016-2018	33	36	100.00%	0
9	Paul, G (34).	2021	Retrospective	Germany	2015-2018	163	42.62	100.00%	40
10	Seña, A. C (35).	2011	Retrospective	US	2000-2009	465	27.1	61.70%	115
11	Smith, N. H (36).	2004	Prospective	US	NR	24	34.96	87.10%	NR
12	Spornraft-Ragaller, P (37).	2011	Retrospective	Germany	2001-2008	24	41	100.00%	0
13	Wang, Z. S (38).	2018	Retrospective	China	2001-2013	70	32	35.70%	1
14	Wu, H (39).	2021	Retrospective	China	2011-2017	276	NR	54.71%	73
15	Zhang, R. L (40).	2017	Prospective	China	2011-2015	514	NR	73.20%	401
16	Zhang, X (41).	2021	Prospective	China	2012-2016	116	35	25.86%	5
17	Tong, M.L (42).	2013	Retrospective	China	2005-2010	1327	41.02	58.90%	292

First author	Secondary syphilis	Early latent syphilis	Tertiary or neurosyphilis	Serofast state definition
Cai, S. N.	NR	NR	139	Serofast status was defined as patients who have been at least six months after initial recommended treatment and regular follow-up, a 4-fold decline in serum RPR titer was not observed even after additional treatment.
Leeyaphan, C.	159	14	0	Nontreponemal titers that neither increased nor decreased ≥4-fold after treatment were in a serofast state.
Liu, J.	58	382	0	Serofast status is defined as a < 4-fold (twofold dilution) decline in non-treponemal antibody titer at 6–12 months or as persistently low titer after treatment.
Liu, Y.	16	88	0	Serofast is defined as either no change in RPR titer or a 2-fold decrease (1-fold dilution) or increase in titer following initial therapy or retreatment.
Marchese, V.	0	386	0	Patients with a persistently reactive RPR titre despite adequate treatment and an initial ≥4-fold decline were defined as serofast.
Pastuszczak, M.	37	46	0	Serofast state group, defined as failure of the RPR titre to decrease ≥4-fold at 6 months after exclusion of neurosyphilis.
Pastuszczak, M.	27	19	0	Noteworthy, we defined the serofast state to be lack of at least a four-fold decline in RPR titer after syphilis treatment.
Pastuszczak, M.	0	20	5	However, a substantial proportion of patients demonstrates <4-fold reduction of titers after recommended therapy (serofast state).
Paul, G.	12	0	0	Serofast state was defined as a less than four-fold decrease in non-treponemal antibody titres during a 6-month follow-up period in the absence of symptoms of syphilis.
Seña, A. C.	218	132	0	Serofast status was defined as either no change in titer or a 2-fold (1 dilution) titer decrease or increase from baseline.
Smith, N. H.	NR	NR	NR	Serofast was defined as a persistent titre of RPR even after syphilis treatment.
Spornraft-Ragaller, P.	21	0	3	NR
Wang, Z. S.	15	54	0	Serofast was defined as either no change in RPR titre or a 1 dilution (2-fold) decrease or increase in titre following initial therapy or retreatment.
Wu, H.	95	108	0	Serofast is defined as either no change in RPR titer or a 2-fold decrease (1-fold dilution) or increase in titer post-treatment.
Zhang, R. L.	113	0	0	Serofast status was defined as either no change in RPR test results or less than a twofold (one dilution) decline or increase in titers at 12 months, as compared with baseline titers.
Zhang, X.	8	7	0	The term”serofast” has been used to refer to patients with syphilis who have “serologic nonresponse”; defined as a <4-fold titer decline after treatment;
Tong, M.L.	360	416	259	After undergoing the recommended therapy, patients with early syphilis did not exhibit two-dilution declines or two-dilution increases in the nontreponemal antibody titre. These individuals were in a ‘‘serofast state’’ one year after treatment.

First author	Main syphilis treatment regimens	Alternative syphilis treatment regimens	Nontreponemal antibody test	No. of serofast patients	Follow-up
Cai, S. N.	Benzathine penicillin	Ceftriaxone, Tetracycline, Erythrosine	RPR	263	12 months
Leeyaphan, C.	Benzathine penicillin	Doxycycline, Ceftriaxone	VDRL	5	12 months
Liu, J.	Benzathine penicillin	NR	RPR	309	12 months
Liu, Y.	Benzathine penicillin	Doxycycline, Procaine penicillin	RPR	76	24 months
Marchese, V.	Benzathine penicillin	NR	RPR	102	12 months
Pastuszczak, M.	Benzathine penicillin	NR	RPR	28	6 months
Pastuszczak, M.	Benzathine penicillin	NR	RPR	10	12 months
Pastuszczak, M.	Benzathine penicillin	NR	RPR	12	12 months
Paul, G.	Benzathine penicillin	Doxycycline	RPR	61	12 months
Seña, A. C.	Benzathine penicillin	Doxycycline, Azithromycin	RPR	96	6 months
Smith, N. H.	Procaine penicillin	Ceftriaxone	RPR	5	32 months
Spornraft-Ragaller, P.	Benzathine penicillin	Ceftriaxone	RPR	1	19 months
Wang, Z. S.	Benzathine penicillin	Erythromycin,Doxycycline,Procaine penicillin	RPR	36	12 months
Wu, H.	Benzathine penicillin	Minocycline	RPR	37	24 months
Zhang, R. L.	Benzathine penicillin	NR	RPR	104	12 months
Zhang, X.	Benzathine penicillin	Doxycycline,Tetracycline,Procaine penicillin	RPR	47	36 months
Tong, M.L.	Benzathine penicillin	Doxycycline, Azithromycin	RPR	457	12 months

NR, not reported; RPR, Rapid Plasma Reagin; VDRL, Venereal Disease Research Laboratory Test.

### Quality assessment

Two authors independently assessed the methodological quality of each qualified study using the Newcastle-Ottawa Scale (NOS) (23). The scale covers three domains, including patient representativeness, exposure and outcome determination, and follow-up adequacy. The total score for each study was 9. A score ≥ 8 indicates high quality (low bias risk) (**Table 2**) (24).

**Table 2:** Methodological quality score of the included studies based on the Newcastle–Ottawa Scale (NOS) tool.

Author	Year	Study design	Selection				Comparability	Exposure/Outcome			Total score	Risk of bias
			Representativeness of cohort *	Selection of control cohort *	Ascertainment of exposure *	Outcome not present at start *	Comparability of cohorts **	Assessment of outcome *	Length of follow-up *	Adequacy of follow-up *	Total score 9*	
Cai, S. N (20).	2017	Retrospective study	*	*	*	*	/	*	*	*	7	High
Leeyaphan, C (27).	2019	Retrospective study	*	/	*	*	**	*	*	*	8	Low
Liu, J (28).	2022	Prospective study	*	*	*	*	*	*	*	*	8	Low
Liu, Y (29).	2020	Retrospective study	*	/	*	*	**	*	*	*	8	Low
Marchese, V (30).	2022	Retrospective study	*	/	*	*	**	*	*	/	7	High
Pastuszczak, M (31).	2019	Prospective study	*	*	*	*	**	*	/	*	8	Low
Pastuszczak, M (32).	2019	Prospective study	*	*	*	*	*	*	*	*	8	Low
Pastuszczak, M (33).	2018	Retrospective study	*	*	*	*	/	*	*	/	6	High
Paul, G (34).	2021	Retrospective study	*	*	*	*	**	*	*	*	9	Low
Seña, A. C (35).	2011	Retrospective study	*	*	*	*	**	*	/	*	8	Low
Smith, N. H (36).	2004	Prospective study	*	*	*	*	**	*	*	*	9	Low
Spornraft-Ragaller, P (37).	2011	Retrospective study	*	*	*	*		*	*	*	8	Low
Wang, Z. S (38).	2018	Retrospective study	*	*	*	*	**	*	*	*	9	Low
Wu, H (39).	2021	Retrospective study	*	*	*	*	**	*	*	*	9	Low
Zhang, R. L (40).	2017	Prospective study	*	*	*	*	**	*	*	*	9	Low
Zhang, X (41).	2021	Prospective study	*	*	*	*		*	*	*	8	Low
Tong, M.L (42).	2013	Retrospective study	*	*	*	*	**	*	*	*	9	Low

*, 1point; **, 2 points.

### Statistical analysis

The data that were extracted from the studies were the correlative factors affecting the syphilis serofast state were statistically analysed using Stata 18.0 software (Stata Corp, College Station, Texas, USA). Fully adjusted effect estimates (RR) for the association in risks of serofast state were used to derive pooled risk estimates, depicted graphically with forest plots. Heterogeneity between studies was assessed by I^2^ statistics. Publication bias was evaluated by visual evaluation of funnel chart symmetry combined with Egger’s test. In addition, we used the adjustment and filling method put forward by Duvall & Twedie to adjust the risk estimates to assess the potential impact of publication bias (25). Sensitivity analysis was carried out by omitting individual studies to evaluate the stability of the results. Heterogeneity between studies was evaluated by using the Cochrane Q test and I² test, I² ≥ 50 or P < 0.05 was considered to indicate statistical significance (26). To reduce the influence of heterogeneity on the outcome, we used a random effects model.

## Results

### Literature search

**Figure 1:**
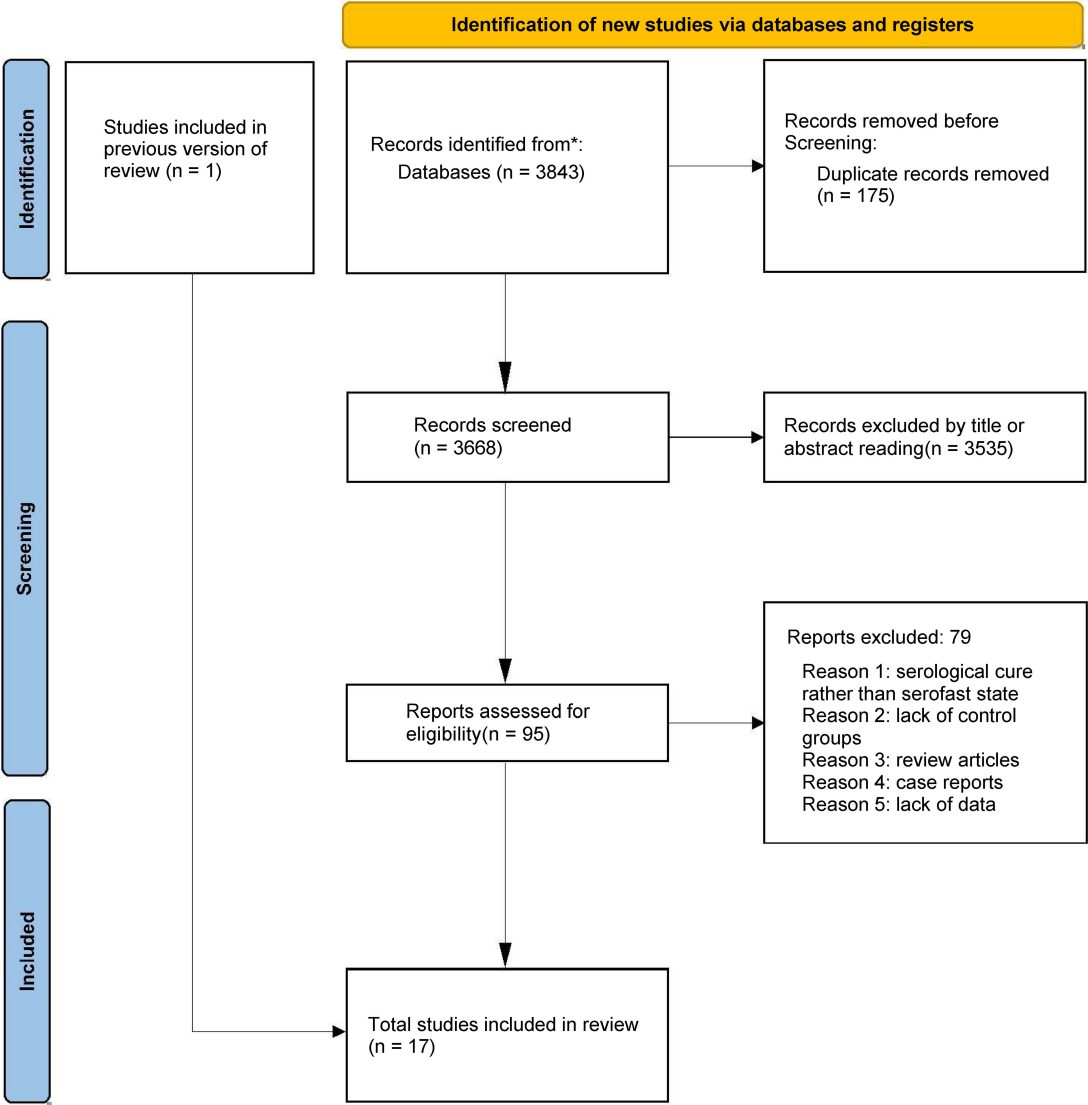
Flowchart of the study selection. The flow diagram illustrates the systematic search and screening process based on PRISMA guidelines. A total of 3,843 records were identified. After removing 175 duplicates, 3,668 articles were screened, and 17 cohort studies meeting the inclusion criteria were finally included in the meta-analysis.

We initially identified 3843 articles through keyword search, and after removing duplicate articles, there were 3668 articles. Through screening of their titles and abstracts, we conducted a full-text review of 95 studies. Ultimately, 17 cohort studies involving 4662 patients met the inclusion criteria and were included in the meta-analysis (20, 27–41), while 79 studies were excluded (**Figure 1**). Among the 79 excluded articles, the reasons for exclusion were serological cure rather than serofast state, lack of control groups, review articles, case reports, and lack of data.

### Baseline characteristics

All included studies were published between 2004 and 2022, and the baseline characteristics are shown in **Table 1**. Eight studies were conducted in China (20, 28, 29, 38–42), three in Poland (31–33), two in the United States (35, 36), two in Germany (34, 37), one in Thailand (27), and one in Italy (30). Most studies (11/17) are retrospective cohort studies, while some studies (6/17) are prospective cohort studies. Nine studies defined a state where the RPR titer of syphilis patients neither increased nor decreased 4−fold after treatment as serofast state, while seven studies defined serofast state as a state where the RPR titer did not change or a 2-fold decrease (1-fold dilution) or increase in titer post-treatment. 82.35% (14/17) of the included studies were high-quality studies with a score of ≥ 8 (**Table 2**).

### Risk factors

#### Gender

**Figure 2:**
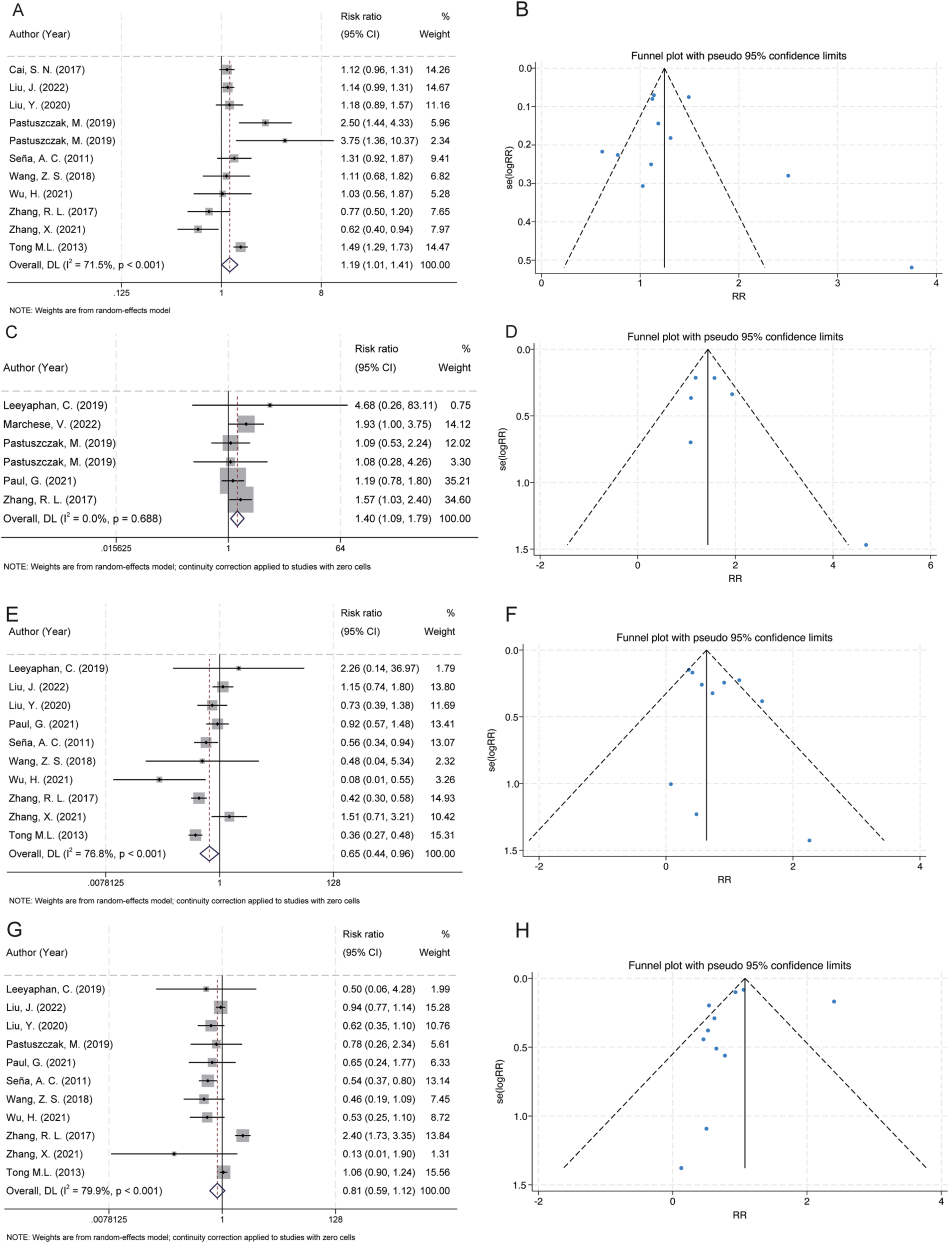
Forest and funnel plots showing the associations between serofast state and gender, HIV infection, and syphilis stage. **(A)** Female gender was significantly associated with an increased risk of serofast state. **(B)** Funnel plot for gender indicates low risk of publication bias. **(C)** HIV coinfection was positively associated with serofast state. **(D)** Funnel plot for HIV shows approximate symmetry. **(E)** Primary syphilis was significantly associated with a decreased risk of serofast state. **(F)** Funnel plot for primary syphilis appears symmetric. **(G)** No significant association was observed between secondary syphilis and serofast state. **(H)** Funnel plot for secondary syphilis appears symmetric.

A total of 11 studies examined the association between gender and serofast state (20, 28, 29, 31, 32, 35, 38–42). The pooled analysis revealed a significant association between female gender and serofast occurrence (RR = 1.19, 95% CI: 1.01–1.41) (**Figure 2A**). Substantial heterogeneity was detected across studies (I² = 71.5%, P < 0.001). Sensitivity analysis demonstrated that the combined RR remained stable upon sequential exclusion of individual studies, with estimates ranging from 1.14 (95% CI: 0.96–1.36) to 1.25 (95% CI: 1.07–1.46) (**Supplementary Table S2**). Visual assessment of the funnel plot indicated general symmetry, suggesting no substantial publication bias (**Figure 2B**). This observation was further supported by both Egger’s test (P = 0.530) and Begg’s test (P = 0.350).

#### HIV

A total of 6 studies examined the association between HIV infection and serofast state (27, 30–32, 34, 40). The pooled analysis demonstrated a positive association between HIV and serofast occurrence (RR = 1.40, 95% CI: 1.09–1.79) (**Figure 2C**). However, no significant heterogeneity was detected across studies (I² = 0.0%, P = 0.688). Sensitivity analysis through sequential exclusion of individual studies revealed stable effect estimates, with RR values ranging from 1.31 (95% CI: 0.97–1.79) to 1.53 (95% CI: 1.12–2.08) (**Supplementary Table S2**). Visual inspection of the funnel plot revealed general symmetry, suggesting low likelihood of publication bias (**Figure 2D**). Nevertheless, formal statistical assessment using Egger’s or Begg’s test was not performed due to the limited number of included studies (n < 10), which precludes reliable detection of small-study effects.

#### Syphilis stages

##### Primary syphilis

A total of 10 studies examined the association between primary syphilis and serofast state (27–29, 34, 35, 38–42). Primary syphilis was significantly associated with a reduced risk of serofast occurrence (RR = 0.65, 95% CI: 0.44–0.96) (**Figure 2E**). Substantial heterogeneity was detected across studies (I² = 76.8%, P < 0.001). Sensitivity analysis demonstrated that the combined RR remained stable upon sequential exclusion of individual studies, with estimates ranging from 0.59 (95% CI: 0.40–0.87) to 0.72 (95% CI: 0.48–1.09) (**Supplementary Table S2**). Visual assessment of the funnel plot indicated general symmetry, suggesting no substantial publication bias (**Figure 2F**). This observation was further supported by both Egger’s test (P = 0.232) and Begg’s test (P = 0.474).

##### Secondary syphilis

A total of 11 studies examined the association between secondary syphilis and serofast state (27–29, 32, 34, 35, 38–42). No significant association was found between secondary syphilis and serofast occurrence (RR = 0.81, 95% CI: 0.59–1.12) (**Figure 2G**). Substantial heterogeneity was detected across studies (I² = 79.9%, P < 0.001). Sensitivity analysis demonstrated that the combined RR remained stable upon sequential exclusion of individual studies, with estimates ranging from 0.74 (95% CI: 0.48–1.14) to 0.87 (95% CI: 0.63–1.22) (**Supplementary Table S2**). Visual assessment of the funnel plot indicated general symmetry, suggesting no substantial publication bias (**Figure 2H**). This observation was further supported by both Egger’s test (P = 0.586) and Begg’s test (P = 0.276).

##### Latent syphilis

**Figure 3:**
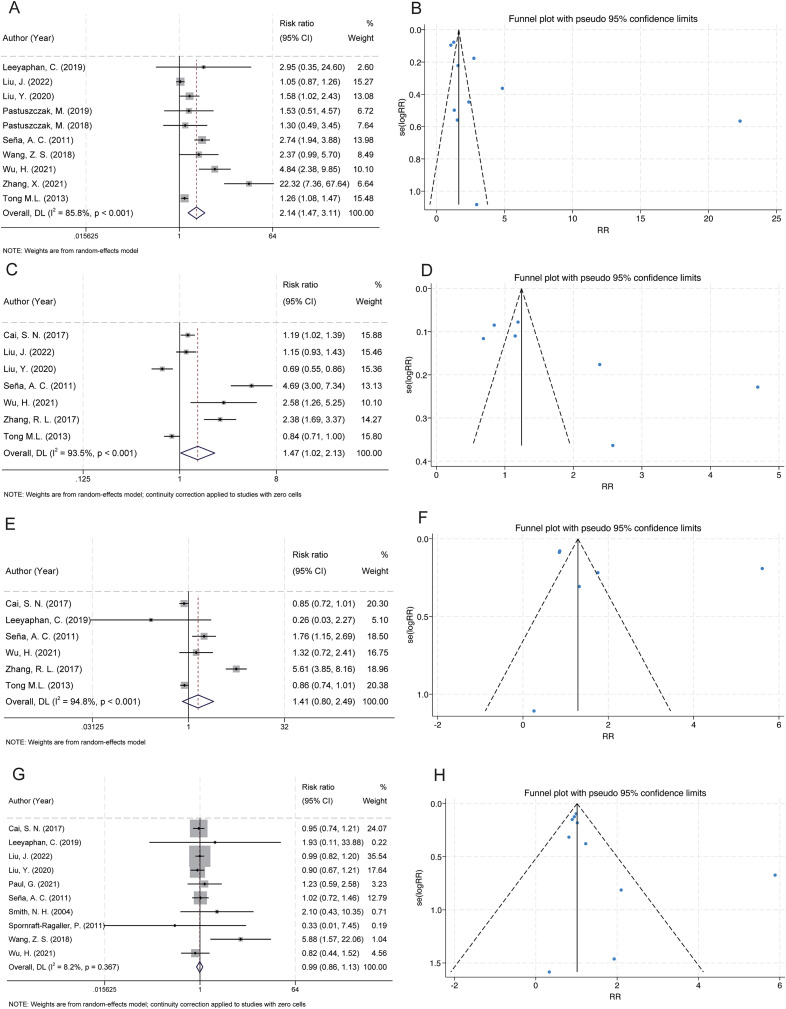
Forest and funnel plots showing the associations between serofast state and latent syphilis, RPR titres, age, and benzathine penicillin treatment. **(A)** Latent syphilis was significantly associated with a higher risk of serofast state. **(B)** Funnel plot for latent syphilis indicates low publication bias. **(C)** RPR titres ≤1:32 were significantly associated with an increased risk of serofast state. **(D)** Funnel plot for RPR titres shows mild asymmetry. **(E)** No significant association was found between age >40 years and serofast state. **(F)** Funnel plot for age appears symmetric. **(G)** Benzathine penicillin treatment showed no significant association with serofast state. **(H)** Funnel plot for benzathine penicillin suggests no evident publication bias.

A total of 10 studies examined the association between latent syphilis and serofast state (27–29, 32, 33, 35, 38, 39, 41, 42). The pooled analysis revealed a significant association between latent syphilis and serofast occurrence (RR = 2.14, 95% CI: 1.47–3.11) (**Figure 3A**). Substantial heterogeneity was detected across studies (I² = 85.8%, P < 0.001). Sensitivity analysis demonstrated that the combined RR remained stable upon sequential exclusion of individual studies, with estimates ranging from 1.76 (95% CI: 1.28–2.42) to 2.50 (95% CI: 1.55–4.01) (**Supplementary Table S2**). Visual assessment of the funnel plot indicated general symmetry, suggesting no substantial publication bias (**Figure 3B**). This observation was further supported by both Egger’s test (P = 0.116) and Begg’s test (P = 0.210).

#### Baseline titers of non-treponemal serological testing: rapid plasma reagin card assay

Several methods can be used to measure the decline in nontreponemal antibody titers, and different testing methods may influence the results of the study. Based on the large body of research available and the widespread use of the RPR test, we selected RPR as the representative test for this factor in our analysis.

A total of 7 studies examined the association between low rapid plasma reagin (RPR) titers (≤1:32) and serofast state (20, 28, 29, 35, 39, 40, 42). The pooled analysis revealed a significant association between low RPR antibody titers (≤1:32) and serofast occurrence (RR = 1.47; 95% CI: 1.02–2.13) (**Figure 3C**). However, substantial between-study heterogeneity was detected (I² = 93.5%, P < 0.001), necessitating cautious interpretation of these findings. Sensitivity analysis demonstrated that the combined RR remained stable upon sequential exclusion of individual studies, with estimates ranging from 1.21 (95% CI: 0.89–1.64) to 1.69 (95% CI: 1.14–2.51) (**Supplementary Table S2**). Visual inspection of the funnel plot revealed an asymmetrical distribution with smaller-scale studies clustering toward the side of diminished effect magnitudes (**Figure 3D**). Formal statistical assessment using Egger’s (P = 0.062) and Begg’s (P = 0.230) tests did not achieve conventional significance thresholds for publication bias detection, though this null finding may reflect limited statistical power inherent to meta-analyses with few included studies (n = 7). The observed asymmetry could alternatively arise from clinical heterogeneity across studies (e.g., differential inclusion of syphilis stages) or random variation rather than selective publication bias. Given the methodological constraints imposed by the limited study pool and substantial heterogeneity, these meta-analytic findings should be considered provisional. Definitive characterization of the RPR-serofast relationship will require large-scale prospective cohort studies employing standardized serological monitoring protocols and rigorous control for potential confounders.

#### Age>40

A total of 6 studies examined the association between advanced age (>40 years) and serofast state (20, 27, 35, 39, 40, 42). No significant association was found between advanced age (>40 years) and serofast occurrence (RR = 1.41; 95% CI: 0.80–2.49) (**Figure 3E**). Substantial heterogeneity was detected across studies (I² = 94.8%, P < 0.001). Sensitivity analysis demonstrated that the combined RR remained stable upon sequential exclusion of individual studies, with estimates ranging from 1.02 (95% CI: 0.79–1.33) to 1.55 (95% CI: 0.87–2.77) (**Supplementary Table S2**). Funnel plot asymmetry assessment revealed no substantial asymmetry through visual inspection, implying a low likelihood of publication bias (**Figure 3F**). However, formal statistical evaluation using Egger’s or Begg’s tests was precluded due to insufficient statistical power (n = 6 studies), as current methodological guidelines recommend a minimum of 10 studies for reliable bias detection through regression-based approaches. The null association observed here may reflect true biological independence, insufficient sample sizes, or residual confounding from unmeasured variables (e.g. treatment adherence differences across age groups). Prospective studies stratifying participants by decade-specific age cohorts could elucidate potential non-linear relationships.

#### Benzathine penicillin

A total of 10 studies examined the association between benzathine penicillin and serofast state (20, 27–29, 34–39). No significant association was found between benzathine penicillin and serofast occurrence (RR = 0.99; 95% CI: 0.86–1.13). However, no significant heterogeneity was detected across studies (I² = 8.2%, P = 0.367) (**Figure 3G**). Sensitivity analysis through sequential exclusion of individual studies revealed stable effect estimates, with RR values ranging from 0.97 (95% CI: 0.86–1.10) to 1.02 (95% CI: 0.86–1.20) (**Supplementary Table S2**). Visual assessment of the funnel plot indicated general symmetry, suggesting no substantial publication bias (**Figure 3H**). This observation was further supported by both Egger’s test (P = 0.161) and Begg’s test (P = 0.210).

## Discussion

### Principal findings

This comprehensive meta-analysis synthesizes evidence from 17 cohort studies investigating determinants of serofast state in syphilis management (20, 27–41). The pooled data demonstrate statistically robust associations between serofast occurrence and key clinical progression parameters, particularly disease stage at diagnosis (primary vs. latent), gender, HIV coinfection status and low RPR antibody titers (≤1:32) (**Table 3**). Notably, these risk relationships exhibited consistently elevated effect magnitudes across most stratified subgroups, with findings remaining robust in sensitivity analyses employing sequential exclusion methodologies. Crucially, our analysis failed to establish significant epidemiological associations between serofast persistence and three widely debated factors: advanced age thresholds (>40 years), secondary syphilis and penicillin-based therapeutic regimens (**Table 3**). The null findings for these variables persisted through multiple analytic frameworks, including random-effects models and influence analyses. We believe that the lack of significant association may reflect differences in study design, follow-up duration, and population characteristics across the included studies. Variations in inclusion criteria, the timing of follow-up, as well as heterogeneity in treatment regimens and patient demographics could account for the discrepancies observed in the literature. Further research with standardized protocols and longer follow-up durations is warranted to better understand the impact of these factors on serofast persistence.

**Table 3:** Significant and nonsignificant risk factors associated with serofast state.

Significant factors	No.studies	No.patients	Serofast state RR (95% CI)	I2, %	P
Gender					
Female	11	2530	1.19 (1.01 - 1.41)	71.50	0.000
HIV					
Positive	6	753	1.40 (1.09 - 1.79)	0.00	0.688
Primary syphilis					
Positive	10	2030	0.65 (0.44 - 0.96)	76.80	0.000
Latent syphilis					
Positive	10	1698	2.14 (1.47 - 3.11)	85.80	0.000
RPR					
≤1:32	7	2631	1.47 (1.02 - 2.13)	93.50	0.000
Therapeutic regimen					
Benzathine penicillin	10	1719	0.99 (0.86 - 1.13)	8.20	0.367
Non-significant factors	No.studies	No.patients	Serofast state RR (95% CI)	I2, %	P value
Age (years)					
>40	6	1687	1.41 (0.80 - 2.49)	94.80	0.000
Secondary syphilis					
Positive	11	2048	0.81 (0.59 - 1.12)	79.90	0.000

### Comparison with other studies

Our investigation into serofast determinants demonstrates concordance with existing systematic reviews regarding the elevated risk associated with primary/latent syphilis stages and female gender. Notably, our findings align with the conclusions of two prior systematic reviews (43, 44), though critical methodological limitations in these earlier studies warrant discussion. The study of Qin, J., et al. (44), while valuable, exhibited constrained generalizability due to its exclusive focus on Chinese populations and failure to establish associations between RPR titers, HIV coinfection, and serofast outcomes. Similarly, Cao, Q., et al.’s analysis lacks essential statistical validation (43), potentially compromising result reliability. This study is currently the most comprehensive one, involving 4662 participants, aiming to conduct a meta-analysis on the relationship between serofast state and multiple outcomes.

### Potential mechanisms

Current understanding of the pathophysiological mechanisms and clinical implications underlying serofast state remains incompletely elucidated (1, 10, 45). Emerging evidence suggests that host immune status and specific *T. pallidum* subtypes may significantly influence post-therapeutic serological outcomes (14). Recent investigations have revealed substantial cellular immunoregulatory disturbances in serofast patients, particularly involving Th1/Th2 cytokine polarization imbalance and Th17/Treg cell proportion dysregulation (46). Quantitative analysis demonstrates significantly elevated Treg cell populations in peripheral blood samples from serofast cases compared with healthy controls, concurrent with markedly reduced Th17 cell frequencies (47). This immunological perturbation may reflect heightened cellular immunosuppression, potentially resulting in suboptimal antibody production and subsequent development of serological persistence (46).

The pathogenic heterogeneity among *T. pallidum* strains may constitute a critical determinant in serological outcomes. Lin, L. R., et al. demonstrated Tp-IgM’s utility as a diagnostic biomarker for syphilis recrudescence and active infection (48). Mechanistically, genomic polymorphism in the Tpr gene family facilitates antigenic diversification, enabling immune evasion through epitope variation and contributing to serological persistence (49). Molecular epidemiological studies by Marra, C., et al. identified strain 14d/f as the predominant circulating variant (50), with distinct subtype distributions correlating with differential seroconversion rates. Notably, serofast cases exhibited higher 14i/a subtype prevalence compared to seroreverted counterparts, suggesting subtype-specific virulence factors may increase the possibility of serofast occurrence (11).

### Implications

Our research may provide valuable insights for future clinical practice, as it assesses the influence of various factors on the occurrence of serum rapid reactions. In the early stages of syphilis, personalized treatment approaches based on individual patient conditions may help reduce the risk of serofast state. The positive association between HIV infection and serofast persistence suggests that HIV-positive individuals may require more intensive or tailored treatment regimens. Closer monitoring of serologic response, including RPR titers, and more frequent follow-up may be particularly beneficial for this subgroup. While there remains some debate about the long-term health effects of serofast state, we believe that its emergence is often underrecognized by clinicians. This research offers additional considerations for clinicians, particularly in managing patients at risk of serofast state, and may help improve treatment strategies, leading to more effective syphilis management in the future.

### Strengths and limitations

This investigation demonstrates three principal methodological strengths in advancing serofast research. First, the relevant articles studied in this meta-analysis are the largest and most comprehensive meta-analysis related to the topic, providing the latest evidence on the potential relationship between primary syphilis, latent syphilis, female gender, HIV, low RPR antibody titers (≤1:32) and serofast state. Second, our meta-analysis covers a wider range, conducting a comprehensive search of literature using subject words and free words, and developing a search strategy without language or date restrictions. In this way, more original articles that meet the inclusion criteria can be found, in order to avoid publication bias and improve the credibility of the results. Third, we adhered to the PRISMA guidelines and used different methods to test the stability of the results, including sensitivity analysis and publication bias test, and the results were consistent with the main analysis.

This study has several methodological limitations that warrant consideration. First, significant heterogeneity was observed among the included studies, which may stem from the absence of standardized diagnostic criteria for serofast state and variations in enrollment protocols, therapeutic regimens, and follow-up schedules across different cohort studies, so we have compiled and organized the differential information that may have an impact on the research conclusions (**Table 1**). Notably, due to the lack of individual patient-level data, we were unable to perform subgroup analyses to investigate potential confounding factors such as therapeutic regimen variations, high-risk sexual behaviours (e.g. homosexual contact), and racial genetic predispositions. Second, given the inherent limitations of observational cohort studies, our findings only establish associations between serofast state and female gender, primary syphilis, latent syphilis, and HIV co-infection, rather than demonstrating causal relationships. To address these methodological constraints, future multicenter prospective cohort studies are warranted. Finally, we assessed the methodological quality of each included study based on three domains: patient representativeness, exposure and outcome determination, and follow-up adequacy. We identified three high-risk studies in our analysis, which could potentially affect the reliability of our conclusions. However, for the sake of data comprehensiveness and other considerations, we chose to retain these three high-risk studies in our analysis. To mitigate the impact of lower-quality studies on the final conclusions, we employed the Newcastle-Ottawa Scale (NOS) for quality assessment and applied appropriate weighting. Additionally, we performed sensitivity analysis through leave-one-out methodology to further ensure the robustness of our results.

## Conclusions

Current evidence indicates that among syphilis patients undergoing treatment, several factors demonstrate association with the serofast phenomenon - specifically female gender, co-existing HIV infection, primary syphilis, latent syphilis, and low RPR antibody titers (≤1:32). However, confirmation of these risk factors and investigation into their modifiability for serofast prevention require validation through prospective large-scale cohort studies.

## Data Availability

The raw data supporting the conclusions of this article will be made available by the authors, without undue reservation.
