# Supplementary material for: Risk factors of serofast state in patients undergoing syphilis: a meta-analysis of 17 cohort studies
Source: Front Immunol. 2025 Dec 5;16:1689904. doi: 10.3389/fimmu.2025.1689904 (PMC12714923; doi:10.3389/fimmu.2025.1689904)
Supplement: Supplementary file 1 [file Table1.docx]

**Table S1**: General Search Strategies for PubMed and Embase.

| **Text S1 Search strategy**  **Database: Pubmed from inception to Present> (Search date: February 20, 2024)**  **Search Strategy:**  --------------------------------------------------------------------------------  ***Syphilis terms(P):***  #1 "Syphilis*"[Mesh]  #2 "Treponema pallidum"[Mesh]  #3 (Syphilis, Latent or Syphilis, Cutaneous or Syphilis, Cardiovascular or Syphilis, Congenital or Neurosyphilis or Chancre) [Title/Abstract]  #4 #1-#3/or  ***Therapy terms(I):***  #5 "Therapeutics"[Mesh]  #6 (Balneology or Ammotherapy or Behavior Control or [Blood Component Removal](https://www.ncbi.nlm.nih.gov/mesh/68001781) or Bridge Therapy or Combined Modality Therapy or Complementary Therapies)[Title/Abstract]  #7 "Drug Therapy"[Mesh]  #8 "[Biological Therapy](https://www.ncbi.nlm.nih.gov/mesh/68001691)"[Mesh]  #9 ([Blood Transfusion](https://www.ncbi.nlm.nih.gov/mesh/68001803) or [Cell- and Tissue-Based Therapy](https://www.ncbi.nlm.nih.gov/mesh/68064987) or [Genetic Therapy](https://www.ncbi.nlm.nih.gov/mesh/68015316) or [Immunomagnetic Separation](https://www.ncbi.nlm.nih.gov/mesh/68018189) or [Immunomodulation](https://www.ncbi.nlm.nih.gov/mesh/68056747) or [Phage Therapy](https://www.ncbi.nlm.nih.gov/mesh/2016365)) [Title/Abstract]  #10 #5-#9/or  ***Results terms(O):***  #11 "Syphilis Serodiagnosis"[Mesh]  #12 (Serodiagnosis, Syphilis or Syphilis Serodiagnoses or Kahn Test or Wassermann Reaction) [Title/Abstract]  #13 (Fluorescent Treponemal Antibody-Absorption Test or Treponema Immobilization Test or Serologic Tests) [Title/Abstract]  #14 (serofast or seroresistance or serology) [Title/Abstract]  #15 "Treatment Outcome"[Mesh]  #16 #11-#15/or  ***Final search results: The effect of electrical stimulation on spinal fusion surgery:***  #17 #4 and #10 and #16(666)  **Text S2 Search strategy**  **Database: EMBASE from inception to Present> (Search date: September 1, 2024)**  **Search Strategy:**  --------------------------------------------------------------------------------  ***Syphilis terms(P):***  #1 'syphilis'/exp  #2 'treponema pallidum'/exp  #3 (‘syphilis, latent’ or ‘syphilis, cutaneous’ or ‘syphilis, cardiovascular’ or ‘syphilis, congenital’ or ‘neurosyphilis’ or ‘chancre’):ab,ti  #4 #1-#3/or  ***Therapy terms(I):***  #5 'therapy'/exp  #6 (‘balneology’ or ‘ammotherapy’ or ‘behavior control’ or ‘[blood component removal](https://www.ncbi.nlm.nih.gov/mesh/68001781)’ or ‘bridge therapy’ or ‘combined modality therapy’ or ‘complementary therapies’):ab,ti  #7 'drug therapy'/exp  #8 'immunotherapy'/exp  #9 #5-#8/or  ***Results terms(O):***  #10 'syphilis serology'/exp  #11 (‘fluorescent treponemal antibody-absorption test’ or ‘treponema immobilization test’ or ‘serologic tests’):ab,ti  #12 (‘serofast’ or ‘seroresistance’ or ‘serology’):ab,ti  #13 'treatment outcome'/exp  #14 #10-#13/or  ***Final search results: The effect of electrical stimulation on spinal fusion surgery:***  #15 #4 and #9 and #14(3177) |
| --- |
