# Supplementary material for: Risk factors of serofast state in patients undergoing syphilis: a meta-analysis of 17 cohort studies
Source: Front Immunol. 2025 Dec 5;16:1689904. doi: 10.3389/fimmu.2025.1689904 (PMC12714923; doi:10.3389/fimmu.2025.1689904)
Supplement: Supplementary file 2 [file Table2.docx]

**Table S2. Results of sensitivity analysis on the basis of outcome assessment.**

| **Study omitted** | **Pooled relative risk (95% CI)** | **Heterogeneity estimate,**  **I^2^, *P* value** |
| --- | --- | --- |
| **Female** |  |  |
| Cai, S. N. 2017 | 1.20 (0.98 - 1.47) | 73.40%, p = 0.000 |
| Liu, J. 2022 | 1.20 (0.98 - 1.48) | 73.50%, p = 0.000 |
| Liu, Y. 2020 | 1.19 (0.99 - 1.44) | 74.30%, p = 0.000 |
| Pastuszczak, M. 2019 | 1.14 (0.97 - 1.34) | 68.20%, p = 0.001 |
| Pastuszczak, M. 2019 | 1.16 (0.99 - 1.36) | 70.30%, p = 0.000 |
| Seña, A. C. 2011 | 1.18 (0.98 - 1.42) | 74.20%, p = 0.000 |
| Wang, Z. S. 2018 | 1.20 (1.00 - 1.43) | 74.20%, p = 0.000 |
| Wu, H. 2021 | 1.20 (1.01 - 1.43) | 74.10%, p = 0.000 |
| Zhang, R. L. 2017 | 1.23 (1.04 - 1.46) | 70.90%, p = 0.000 |
| Zhang, X. 2021 | 1.25 (1.07 - 1.46) | 64.00%, p = 0.003 |
| Tong, M. L. 2013 | 1.14 (0.96 - 1.36) | 63.80%, p = 0.003 |
| **overall** | 1.19 (1.01 - 1.41) | 71.50%, p = 0.000 |
|  |  |  |
| **Age>40** |  |  |
| Cai, S. N. 2017 | 1.51 (0.62 - 3.65) | 95.40%, p = 0.000 |
| Leeyaphan, C. 2019 | 1.55 (0.87 - 2.77) | 95.80%, p = 0.000 |
| Seña, A. C. 2011 | 1.34 (0.70 - 2.56) | 95.60%, p = 0.000 |
| Wu, H. 2021 | 1.43 (0.75 - 2.71) | 95.80%, p = 0.000 |
| Zhang, R. L. 2017 | 1.02 (0.79 - 1.33) | 69.00%, p = 0.012 |
| Tong, M. L. 2013 | 1.50 (0.62 - 3.65) | 95.30%, p = 0.000 |
| **overall** | 1.41 (0.80 - 2.49) | 94.80%, p = 0.000 |
|  |  |  |
| **BP** |  |  |
| Cai, S. N. 2017 | 1.01 (0.84 - 1.22) | 17.30%, p = 0.289 |
| Leeyaphan, C. 2019 | 0.99 (0.85 - 1.15) | 16.60%, p = 0.295 |
| Liu, J. 2022 | 1.00 (0.82 - 1.22) | 18.30%, p = 0.280 |
| Liu, Y. 2020 | 1.02 (0.86 - 1.20) | 14.30%, p = 0.314 |
| Paul, G. 2021 | 0.98 (0.85 - 1.14) | 15.40%, p = 0.305 |
| Seña, A. C. 2011 | 0.99 (0.84 - 1.17) | 18.00%, p = 0.282 |
| Smith, N. H. 2004 | 0.98 (0.86 - 1.13) | 10.50%, p = 0.348 |
| Spornraft-Ragaller, P. 2011 | 0.99 (0.86 - 1.15) | 14.30%, p = 0.315 |
| Wang, Z. S. 2018 | 0.97 (0.86 - 1.10) | 0.00%, p = 0.951 |
| Wu, H. 2021 | 1.00 (0.86 - 1.17) | 15.20%, p = 0.307 |
| **overall** | 0.99 (0.86 - 1.13) | 8.20%, p = 0.367 |
|  |  |  |
| **HIV** |  |  |
| Leeyaphan, C. 2019 | 1.38 (1.08 - 1.78) | 0.00%, p = 0.663 |
| Marchese, V. 2022 | 1.32 (1.01 - 1.73) | 0.00%, p = 0.736 |
| Pastuszczak, M. 2019 | 1.45 (1.11 - 1.88) | 0.00%, p = 0.634 |
| Pastuszczak, M. 2019 | 1.41 (1.09 - 1.81) | 0.00%, p = 0.568 |
| Paul, G. 2021 | 1.53 (1.12 - 2.08) | 0.00%, p = 0.704 |
| Zhang, R. L. 2017 | 1.31 (0.97 - 1.79) | 0.00%, p = 0.623 |
| **overall** | 1.40 (1.09 - 1.79) | 0.00%, p = 0.688 |
|  |  |  |
| **Primary syphilis** |  |  |
| Leeyaphan, C. 2019 | 0.63 (0.42 - 0.95) | 78.80%, p = 0.000 |
| Liu, J. 2022 | 0.59 (0.40 - 0.87) | 70.30%, p = 0.001 |
| Liu, Y. 2020 | 0.64 (0.41 - 0.99) | 79.00%, p = 0.000 |
| Paul, G. 2021 | 0.61 (0.40 - 0.95) | 76.60%, p = 0.000 |
| Seña, A. C. 2011 | 0.66 (0.42 - 1.04) | 79.40%, p = 0.000 |
| Wang, Z. S. 2018 | 0.65 (0.43 - 0.98) | 79.40%, p = 0.000 |
| Wu, H. 2021 | 0.69 (0.47 - 1.02) | 77.10%, p = 0.000 |
| Zhang, R. L. 2017 | 0.70 (0.44 - 1.11) | 77.00%, p = 0.000 |
| Zhang, X. 2021 | 0.59 (0.40 - 0.87) | 74.80%, p = 0.000 |
| Tong, M. L. 2013 | 0.72 (0.48 - 1.09) | 69.00%, p = 0.001 |
| **overall** | 0.65 (0.44 - 0.96) | 76.80%, p = 0.000 |
|  |  |  |
| **Secondary syphilis** |  |  |
| Leeyaphan, C. 2019 | 0.82 (0.59 - 1.14) | 81.70%, p = 0.000 |
| Liu, J. 2022 | 0.76 (0.50 - 1.16) | 81.70%, p = 0.001 |
| Liu, Y. 2020 | 0.84 (0.59 - 1.18) | 80.80%, p = 0.000 |
| Pastuszczak, M. 2019 | 0.81 (0.58 - 1.14) | 81.80%, p = 0.000 |
| Paul, G. 2021 | 0.82 (0.59 - 1.15) | 81.60%, p = 0.000 |
| Seña, A. C. 2011 | 0.87 (0.63 - 1.22) | 77.10%, p = 0.000 |
| Wang, Z. S. 2018 | 0.85 (0.61 - 1.19) | 80.70%, p = 0.000 |
| Wu, H. 2021 | 0.85 (0.61 - 1.19) | 80.80%, p = 0.000 |
| Zhang, R. L. 2017 | 0.74 (0.58 - 0.94) | 53.20%, p = 0.023 |
| Zhang, X. 2021 | 0.83 (0.60 - 1.15) | 81.00%, p = 0.000 |
| Tong, M. L. 2013 | 0.74 (0.48 - 1.14) | 81.50%, p = 0.000 |
| **overall** | 0.81 (0.59 - 1.12) | 79.90%, p = 0.000 |
|  |  |  |
| **Latent syphilis** |  |  |
| Leeyaphan, C. 2019 | 2.12 (1.45 - 3.10) | 87.30%, p = 0.000 |
| Liu, J. 2022 | 2.50 (1.55 - 4.01) | 84.30%, p = 0.000 |
| Liu, Y. 2020 | 2.26 (1.49 - 3.45) | 87.30%, p = 0.000 |
| Pastuszczak, M. 2019 | 2.20 (1.48 - 3.25) | 87.40%, p = 0.000 |
| Pastuszczak, M. 2018 | 2.24 (1.50 - 3.32) | 87.40%, p = 0.000 |
| Seña, A. C. 2011 | 2.02 (1.37 - 2.96) | 82.90%, p = 0.000 |
| Wang, Z. S. 2018 | 2.12 (1.43 - 3.14) | 87.10%, p = 0.000 |
| Wu, H. 2021 | 1.92 (1.32 - 2.77) | 84.40%, p = 0.000 |
| Zhang, X. 2021 | 1.76 (1.28 - 2.42) | 79.50%, p = 0.000 |
| Tong, M. L. 2013 | 2.47 (1.48 - 4.18) | 86.80%, p = 0.000 |
| **overall** | 2.14 (1.47 - 3.11) | 85.80%, p = 0.000 |
|  |  |  |
| **RPR** |  |  |
| Cai, S. N. 2017 | 1.56 (0.96 - 2.54) | 94.50%, p = 0.000 |
| Liu, J. 2022 | 1.56 (1.00 - 2.44) | 94.60%, p = 0.000 |
| Liu, Y. 2020 | 1.69 (1.14 - 2.51) | 93.10%, p = 0.000 |
| Seña, A. C. 2011 | 1.21 (0.89 - 1.64) | 90.20%, p = 0.000 |
| Wu, H. 2021 | 1.38 (0.94 - 2.02) | 94.30%, p = 0.000 |
| Zhang, R. L. 2017 | 1.35 (0.93 - 1.95) | 93.10%, p = 0.000 |
| Tong, M. L. 2013 | 1.65 (1.06 - 2.57) | 93.60%, p = 0.000 |
| **overall** | 1.47 (1.02 - 2.13) | 93.50%, p = 0.000 |

Abbreviations:
